# Epidemiology and outcome of *Staphylococcus aureus* bloodstream infection and sepsis in a Norwegian county 1996–2011: an observational study

**DOI:** 10.1186/s12879-015-0849-4

**Published:** 2015-03-04

**Authors:** Julie Paulsen, Arne Mehl, Åsa Askim, Erik Solligård, Bjørn Olav Åsvold, Jan Kristian Damås

**Affiliations:** Centre of Molecular Inflammation Research, Department of Cancer Research and Molecular Medicine, Norwegian University of Science and Technology, Trondheim, Norway; Department of Medicine, Levanger Hospital, Nord-Trøndelag Health Trust, Levanger, Norway; Department of Circulation and Medical imaging, Norwegian University of Science and Technology, Trondheim, Norway; Clinic of Anaesthesia and Intensive Care, St. Olavs Hospital, Trondheim University Hospital, Trondheim, Norway; Department of Public Health, Norwegian University of Science and Technology, Trondheim, Norway; Department of Infectious diseases, St Olavs Hospital, Trondheim University Hospital, Trondheim, Norway; Department of Endocrinology, St Olavs Hospital, Trondheim University Hospital, Trondheim, Norway; Faculty of Medicine, Institute of Cancer Research and Molecular Medicine, Po box 8905, N-7491 Trondheim, Norway

**Keywords:** *Staphylococcus aureus*, Bacteremia, Sepsis, Organ failure, Comorbid disease, Focus of infection

## Abstract

**Background:**

*Staphylococcus aureus* is one of the most common and lethal causes of bloodstream infection and the incidence is increasing. We carried out a prospective observational study of patients with *Staphylococcus aureus* bloodstream infection and sepsis in Nord-Trøndelag county in Norway from 1996–2011. The main outcome of interest was all-cause mortality within 30 and 90 days.

**Methods:**

Positive blood cultures were registered prospectively by the microbiology laboratory and clinical variables were retrospectively registered from patients’ hospital records. The severity of sepsis was assigned according to the 2001 International Sepsis Definition Conference criteria. The association between clinical characteristics and mortality was studied using logistic regression analysis, and adjusted 30- and 90-day mortality risks were estimated.

**Results:**

Among 373 patients, the median age was 74 years and 60.3% were male. 0.8% of the patients were diagnosed with MRSA. 29.8% of the patients developed severe sepsis and 12.9% developed septic shock. The all-cause mortality was 14.5%, 27.3% and 36.2% at 7, 30 and 90 days, respectively. Compared to patients with sepsis without organ failure (Mortality risk 13.3%, 95% CI 7.5-16.3%), the 30-day mortality risk was 3-fold higher among those with severe sepsis (39.9%, 95% CI 29.5-48.5%) and more than 4-fold higher for those with septic shock (57.3%, 95% CI 42.5-72.2%). The 30-day all-cause mortality varied by focus of infection, with the highest 30-day mortality risk among those with a pulmonary focus (42.4%, 95% CI 26.0-58.5%) and unknown focus of infection (38.7%, 95% CI 27.5-48.2%). The mortality risk did not differ between the first and second halves of the study period with a 30-day mortality risk of 27.3%, (95% CI 18.1-33.1%) for 1996–2003 versus 27.4% (95% CI 19.4-31.4%) for 2004–2011. The same pattern was seen for 90-day mortality risk.

**Conclusion:**

*Staphylococcus aureus* bloodstream infection carries a high case fatality rate, especially among those with severe sepsis and septic shock and among those with a pulmonary or unknown focus of infection. There was no decrease in 30- or 90-day mortality risk during the study period. This underscores the importance of continuing surveillance and efforts to improve the outcome of this serious disease.

**Electronic supplementary material:**

The online version of this article (doi:10.1186/s12879-015-0849-4) contains supplementary material, which is available to authorized users.

## Background

*Staphylococcus aureus* is one of the most lethal and common causes of bloodstream infection, with an incidence of 26/100 000 population/year [1]. Of concern, a 34% increase in incidence has been observed in Europe from 2002–2009 [2]. Factors contributing to the role of *Staphylococcus aureus* as a public health problem include its affinity to foreign objects such as intravenous lines and prosthetic material, and its propensity to generate metastatic foci and complicated disease [3]. Another challenge is its ability to quickly develop resistance to antimicrobial agents [4]. Even in populations with a low level of antibiotic resistance, *Staphylococcus aureus* is a cause of severe bloodstream infection with high mortality [5]. Despite improvements in survival over the last three decades, the 30 day all-cause mortality rates are still at 17-39% [5-10].

Several studies have shown that the outcome of *Staphylococcus aureus* bloodstream infection may differ by focus of infection, with unidentified focus, respiratory focus and endocarditis being associated with the highest mortality [8]. In addition, an uneradicated or non-eradicable focus has been associated with increased mortality [11,12]. Older age, increasing number and types of comorbid diseases before the onset of infection and clinical severity of the bloodstream infection have also been associated with reduced survival [13].

The clinical characteristics and outcome of *Staphylococcus aureus* bloodstream infection are well described in many Western countries [8,14]. However, it is important to study the clinical outcome of bloodstream infections in multiple populations and at multiple time points both to evaluate differences in disease characteristics between populations and to gauge the development over time. It is important to investigate the characteristics of the disease in order to identify areas where management can be improved. Internationally, there has been an increasing effort to improve the management and outcome of sepsis including bloodstream infection over the last decades with initiatives such as the international Surviving Sepsis Campaign [15,16]. In order to improve follow up and treatment of this patient group we carried out a prospective observational study of *Staphylococcus aureus* bloodstream infection in Nord-Trøndelag County.

## Methods

### Setting and population

Nord-Trøndelag is a county in Central Norway with a current population of 134 864. It is served by two community hospitals, Namsos Hospital and Levanger Hospital. The closest tertiary referral hospital is St Olavs University Hospital in Trondheim. We included all patients ≥ age 16 diagnosed with *Staphylococcus aureus* bloodstream infection at Levanger Hospital between 1996 and 2011, and at Namsos Hospital between 1999 and 2011. For residents of Nord-Trøndelag who in 1995–97 had participated in a population survey (the HUNT2 survey) we could also include infections detected at St. Olavs Hospital between 1996 and 2011. All adults in Nord-Trøndelag were invited to the survey and 69.5% participated [17]. All positive blood cultures have been prospectively registered by the clinical microbiology laboratory in Levanger, Namsos and at St. Olavs Hospital. BACTEC 9240 (Becton Dickinson Diagnostic Instrument Systems, Sparks, MD) was used for blood culture testing [18]. Resistance testing was performed by disc diffusion. Methicillin resistance was tested with a cefoxitin disc. Oxacillin-resistant isolates were sent to St Olavs Hospital for testing of the mecA gene.

### Patient characteristics

Clinical information was gathered retrospectively from the patients’ hospital records. All data were collected using a standardized data retrieval form assessing patient characteristics, comorbid conditions, results of investigations and treatment. The data collection was carried out by trained research nurses and all registered data was secondarily assessed either by an infectious disease consultant or the first author of this study. An episode of bloodstream infection was defined as the presence of one or more microorganism(s) in blood culture along with clinical evidence of infection. If a patient had more than one episode of *Staphylococcus aureus* bacteremia during the study period, only the first was included. We decided to include patients with polymicrobial infection in this study since a bloodstream infection containing *Staphylococcus aureus* should be regarded as clinically significant [19].

The setting of infection was classified as hospital-acquired (HA), healthcare-associated (HCA) or community-acquired (CA) as defined by Friedman et al. [20], with the exception that only patients that were hospitalized for two or more days in the 30, as opposed to 90 days prior to the infection were classified as having a HCA infection, in keeping with the definition used by Shorr et al. [21]. The number and severity of combined comorbid conditions were assessed according to the Charlson weighted Comorbidity Index (CCI) [22]. Mortality was measured as all-cause mortality within day 7, 30 and 90. By using the 11-digit unique identification number of all Norwegian citizens, electronic hospital records in Norway are updated with mortality data from the Norwegian population registry so that mortality data after discharge from hospital can be reliably assessed.

### Severity of disease

We graded the severity of disease (sepsis, severe sepsis and septic shock) according to the 2001 International Sepsis Conference definition [15]. In our cohort we defined sepsis as documented bloodstream infection and two or more of the following: temperature ≥38.3°C or < 36.0°C, heart rate >90 beats/minute, respiratory rate >20/min or PaCO_2_ < 4.3 kPa or mechanical ventilation due to acute respiratory failure, glucose >7.7 mmol/l in the absence of diabetes, leucocytes >12 × 10^9^/l or < 4 × 10^9^/l, elevated CRP or procalcitonin, acute hypotension (systolic BT <90 mmHg, MAP <70 mmHg or a fall of ≥ 40 mmHg), or significant positive fluid balance (>20 ml/kg over 24 hours).

Severe sepsis was defined as sepsis and sepsis-related dysfunction in at least one organ, or hypoperfusion or hypotension. Acute organ dysfunction was defined as mental confusion, arterial hypoxemia, acute oliguria, increase in serum creatinine of ≥ 45 μmol/l, low platelet count, coagulation disturbance, ileus, or hyperbilirubinemia. The specific criteria for organ dysfunction used were the same as those outlined in the 2001 International Sepsis Conference definition [15].

Septic shock was defined as sepsis and systolic blood pressure <90 mmHg or a fall in systolic blood pressure of at least 40 mmHg despite at least 1000 ml fluid resuscitation or vasopressor needed to maintain systolic blood pressure >90 mmHg and evidence of organ failure.

Any alteration in disease severity was registered, and the timing and extent of the most pronounced degree of organ dysfunction was noted and used to define whether the patient had sepsis, severe sepsis or septic shock. For those who did not show any evidence of deterioration during the episode, the day of positive blood culture was registered as the day with the most severe affection. Severity of disease was also assessed by the Pitt bacteremia score [23].

### Focus of infection

Reported signs of infection along with focal growth of the same microbe as in blood culture was taken as a confirmation of skin, soft tissue, joint or surgical infection. Respiratory focus was diagnosed with clinical signs of respiratory infection accompanied by positive radiologic findings. Intravenous line infection was diagnosed with growth of *Staphylococcus aureus* from the tip of the catheter as well as in peripheral blood, and also if the microbe was isolated from pus around the catheter entry site. If clinical signs of catheter infections were present without local growth and no other focus was detected an intravenous line infection was registered as likely. *Staphylococcus aureus* has also been shown to be a likely ascending urinary pathogen among patients with urinary tract catheterization or manipulation [24,25]. A urinary focus was assigned when there was growth of bacteria in the urine as well as in blood along with clinical signs/symptoms or risk factor for urinary infection, and no other source of infection was identified. Endocarditis was registered if diagnosed according to clinical and echocardiographic criteria during the hospital stay. An unknown focus of infection was assigned when none of the criteria for ascertaining a focus were met.

### Management

Empiric antibiotic treatment was defined as treatment given before the pathogen was known. Definitive treatment was defined as treatment administered after result of the blood cultures was available. Inefficient antibiotic treatment was defined as treatment to which the microbe was resistant. Treatment was defined as efficient as long as the microbe was sensitive, even if the drug administered was not the first choice.

### Ethical considerations

The study was approved by the Central Norway Regional Committee for Medical Health and Research Ethics. Since no patient contact or intervention was carried out, the need for informed consent was waived.

### Statistical analysis

Data was analyzed using SPSS for Windows (Version 21, Armonk, NY: IBM Corp) and STATA version 13 (StataCorp LP, College Station, Texas). The associations between clinical characteristics and 30- and 90-day mortality were investigated using logistic regression analysis where odds ratios (ORs) with 95% confidence intervals (CIs) were estimated. In addition, we studied the association between clinical characteristics and severe sepsis/septic shock as an outcome, in order to identify groups of patients particularly vulnerable to develop severe disease and need a higher level of follow-up and care. All associations were estimated both unadjusted and adjusted for potential confounders. Thus, all analyses were adjusted for sex and age group (<60, 60–69, 70–79 and ≥80 years). For place of acquisition, focus, severity and time period, the associations were additionally adjusted for prior comorbid conditions using three categories of the CCI (0, 1–2 and ≥3). For the association between individual comorbid conditions and 30- and 90-day mortality, those not having the condition in question were used as reference. Adjusted 30- and 90-day mortality risks and risk of severe sepsis/shock were estimated from the logistic regression model. For ordinal variables, we tested for linear trend across categories by using the categories as a continuous variable in the logistic regression analysis.

## Results

We identified 402 episodes of *Staphylococcus aureus* bloodstream infection during the study period, of which 23 were recurrent episodes. Six patients did not meet the clinical criteria for sepsis, either because of a mild clinical course with only one elevated inflammatory parameter or because clinical or laboratory data needed to assess whether these patients met the sepsis criteria were lacking. For this reason we chose to exclude these patients thus leaving 373 patients for further analysis. 25 patients (6.7%) had polymicrobial infection. 3 patients (0.8%) were diagnosed with MRSA, 2 of these were in the setting of a polymicrobial infection and all three episodes were healthcare-associated. Data on antibiotic susceptibility were available for the 348 monomicrobial isolates. 26.7% were penicillin-sensitive, 99.4% were dicloxacillin-sensitive, 98.5% were gentamicin-sensitive (Table 1).

**Table 1:** **Patient and infection characteristics**

**Characteristic**	**N** (**%)**
Patients included	373 (100)
**Sex**	
Female	148 (39.7)
Male	225 (60.3)
**Acquisition**	
Community-acquired	108 (29.0)
Healthcare-associated	156 (41.8)
Hospital-acquired	109 (29.2)
**Age category**	
<60	81 (21.7)
60-69	58 (15.6)
70-79	109 (29.2)
≥80	125 (33.5)
**Comorbid conditions**	
No underlying illness	20 (5.4)
Malignancy	96 (25.7)
Renal failure	45 (12.1)
Diabetes mellitus	72 (19.3)
Hypertension	114 (30.6)
Cardiovascular disease	154 (41.3)
Heart failure	40 (10.7)
Chronic pulmonary disease	62 (16.6)
Rheumatic disease	39 (10.5)
**Charlson Comorbidity Index (CCI)**	
0	85 (22.8)
1-2	160 (42.9)
≥3	128 (34.3)
**Focus of infection**	
Unknown	94 (25.2)
Respiratory focus	37 (9.9)
Urinary tract	33 (8.8)
Skin/soft tissue	73 (19.6)
Abscess	27 (7.2)
IV catheter	28 (7.5)
Endocarditis	18 (4.8)
Osteomyelitis/Septic arthritis	44 (11.8)
Other*	19 (5.1)
**Microbiological characteristics**	
Polymicrobial infection	25 (6.7)
Methicillin-resistant isolates	3 (0.8)
Penicillin-sensitive isolates§	93 (26.7)
Dicloxacillin-sensitive isolates§	346 (99.4)
Gentamicin-sensitive isolates§$	323 (98.5)
Gentamicin intermediate or resistant§$	5 (1.5)
**Time period**	
1996-2003	144 (38.6)
2004-2011	229 (61.4)

*Other focus includes dental foci, parotitis, 2 cases of meningitis, mediastinits, an infected peritoneal dialysis catheter and one probable intravascular graft infection.

§assessed for monomicrobial episodes, n = 348.

$Data lacking for 20 isolates (5.7%).

The median patient age was 74 years, 60.3% of the cases were male. Overall, 41.8% acquired the infection in a healthcare-associated setting (HCA), 29.2% were hospital-acquired (HA) and 29.0% were community-acquired (CA). Previously known comorbid illness was present in 94.6% of the patients (Table 1). The all-cause mortality rate was 14.5%, 27.3% and 36.2% at 7, 30 and 90 days, respectively (Table 2).

**Table 2:** **Patient outcomes and management**

**Severity of sepsis**	**N (%)**
Sepsis without organ failure	214 (57.4)
Severe sepsis	111 (29.8)
Septic shock	48 (12.9)
**Pitt bacteremia score**	
0	127 (34.0)
1	115 (30.8)
2	65 (17.4)
≥3	66 (17.7)
**All-cause mortality**	
7-day	54 (14.5)
30-day	102 (27.3)
90-day	135 (36.2)
**Empiric antibiotic management**	
Penicillin plus aminoglycoside	65 (17.3)
Cefuroxime-containing regimens	63 (16.9)
Dicloxacillin-containing regimens	62 (16.6)
Penicillin monotherapy	52 (13.9)
Cefotaxime-containing regimens	47 (12.7)
Other treatment	73 (19.6)
No treatment	11 (2.9)
Inefficient treatment	57 (15.3)
Definitive antibiotic treatment*	
Penicillin plus aminoglycoside	18 (4.8)
Dicloxacillin-containing regimens	190 (51.0)
Penicillin monotherapy	34 (9.1)
Cefuroxime-containing regimens	29 (7.9)
Cefotaxime-containing regimens	28 (7.6)
Other treatment	62 (16.7)
No treatment	10 (2.7)
Inefficient treatment	11 (2.9)
**Median antibiotic treatment duration (interquartile range)$**	
Intravenous treatment (N = 351)	10 days (5–15)
Total antibiotic treatment duration (N = 331)	14 days (10–25)
Aminoglycoside treatment (N = 117)	3 days (2–7)
**Other management**	
Received surgical treatment	119 (31.9)
Treatment in Intensive Care Unit (ICU)/High dependency unit	92 (24.7)
Treatment with vasopressors	39 (10.5)
Ventilator treatment	15 (4)
Examined with echocardiography	134 (35.9)
Median length of stay (interquartile range)	13 days (7–22)

*Data on definitive treatment lacking for two patients.

$Data is lacking on 22 patients for intravenous treatment duration and on 42 patients for total antibiotic treatment duration.

The 30-day mortality risk differed by age and by number and severity of comorbid conditions prior to the onset of infection. Compared to those having CCI score of 0 (30-day mortality risk 13.7%, 95% CI 7.0-21.8%), the 30-day mortality risk was 2-fold higher among those with CCI score 1–2 (24.8%, 95% CI 17.6-31.0%) and 3-fold higher for those with CCI score of 3 or more (39.1%, 95% CI 30.0-47.6%) (Table 3). The number and severity of comorbid conditions were similarly associated with 90-day mortality risk (Additional file 1: Table S1).

**Table 3:** **30-day mortality in relation to patient characteristics prior to infection**

			**Age- and sex-adjusted**				
**Characteristic**	**No. of deaths within 30 days**	**30-day mortality within category (%)**	**Odds ratio**	**95% CI**	**p**	**Mortality risk (%)**	**95% CI (%)**
**Age (years)**							
<60	11	13.6	1	Reference		13.6	7.7-23.0
60-69	15	25.9	2.21	0.93-5.26	0.07	25.9	16.2-38.6
70-79	27	24.8	2.09	0.97-4.52	0.06	24.8	17.6-33.8
≥80	49	39.4	4.04	1.95-8.40	<0.001	38.9	30.8-47.8
p for trend					<0.001		
**Sex**							
Male	57	25.3	1	Reference		25.9	19.4-30.9
Female	45	30.4	1.21	0.75-1.94	0.43	29.5	21.6-36.4
**Charlson Comorbidity Index (CCI)**							
0	11	12.9	1	Reference		13.7	7.0-21.8
1-2	41	25.6	2.13	1.02-4.48	0.05	24.8	17.6-31.0
≥3	50	39.1	4.29	2.01-9.14	<0.001	39.1	30.0-47.6
p for trend					<0.001		
**Comorbidities$**							
Malignant disease	26	27.1	0.97	0.57-1.66	0.91	26.9	17.8-35.5
Renal failure	19	42.2	2.18	1.11-4.27	0.02	41.5	27.1-56.3
Diabetes mellitus	21	29.2	0.99	0.56-1.80	>0.99	27.3	17.2-37.5
Hypertension	36	31.6	1.15	0.69-1.91	0.6	29.1	20.3-37.3
Cardiovascular disease	51	33.1	1.29	0.78-2.11	0.32	30.0	22.0-37.2
Heart failure	20	50.0	2.4	1.21-4.80	0.01	43.8	28.7-59.5
Chronic pulmonary disease	25	40.3	2.01	1.12-3.62	0.02	39.2	27.0-51.4
Rheumatic disease	15	38.5	1.84	0.90-3.78	0.09	38.5	23.6-54.3

$Those not having the condition in question were used as reference category for each individual comorbidity studied in this analysis.

57.4% of the patients had sepsis with no organ failure, 29.8% had severe sepsis and 12.9% had septic shock (Table 2). Compared to those with sepsis without organ failure (30-day mortality risk 13.3%, 95% CI 7.5-16.3%), the 30-day mortality risk was 3-fold higher among those with severe sepsis (39.9%, 95% CI 29.5-48.5%) and more than 4-fold higher for those with septic shock (57.3%, 95% CI 42.5-72.2%) (Table 4). A similar association was seen with 90-day mortality risk (Additional file 1: Table S2). A steady increase in mortality risk according to severity was also observed for the Pitt bacteremia score.

**Table 4:** **30-day mortality in relation to disease acquisition, severity, focus and time period**

			**Age-, sex- and comorbidity-adjusted**				
**Characteristic**	**No. of deaths within 30 days**	**30-day mortality within category (%)**	**Odds ratio**	**95% CI**	**p**	**Mortality risk (%)**	**95% CI**
**Place of acquisition**							
Community-acquired	22	20.4	1	Reference		24.2	14.3-31.5
Healthcare-associated	48	30.8	1.18	0.62-2.23	0.61	27.1	18.2-32.5
Hospital-acquired	32	29.4	1.42	0.72-2.78	0.31	30.5	20.2-37.9
**Severity**							
Sepsis without organ failure	27	12.6	1	Reference		13.3	7.5-16.3
Severe sepsis	45	40.5	4.97	2.77-8.93	<0.001	39.9	29.5-48.5
Septic shock	30	62.5	10.98	5.16-23.35	<0.001	57.3	42.5-72.2
p for trend					<0.001		
**Pitt bacteremia score**							
0	26	20.5	1	Reference		20.3	12.0-25.7
1	22	19.1	0.97	0.49-1.88	0.92	19.8	11.3-25.6
2	21	32.3	2.30	1.13-4.79	0.02	35.5	22.6-46.7
≥3	33	50.0	3.66	1.85-7.23	<0.001	45.1	31.9-57.3
p for trend					<0.001		
**Focus of infection**							
Unknown	40	42.6	1	Reference		38.7	27.5-48.2
Respiratory focus	16	43.2	1.18	0.52-2.70	0.69	42.4	26.0-58.5
Urinary tract	4	12.1	0.18	0.05-0.57	0.004	11.2	3.4-24.0
Skin/soft tissue	21	28.8	0.57	0.29-1.14	0.11	27.6	16.5-37.1
Abscess	3	11.1	0.26	0.07-1.00	0.05	15.6	4.3-35.4
Intravenous catheter	6	21.4	0.37	0.13-1.05	0.06	20.3	7.8-36.3
Endocarditis	2	11.1	0.22	0.04-1.07	0.06	13.4	2.8-37.4
Osteomyelitis/Septic arthritis	5	11.4	0.23	0.08-0.67	0.007	13.8	4.9-26.1
Other focus	5	26.3	0.63	0.2-2.0	0.43	29.4	11.4-52.2
**Time period**							
1996-2003	35	24.3	1	Reference		27.3	18.1-33.1
2004-2011	67	29.3	1.00	0.60-1.67	0.99	27.4	19.4-31.4

The focus of infection was ascertained in 74.5% of the patients, whereas in 25.5%, no focus was identified. The most common focus was skin and soft tissue infections (19.6%), whereas 4.8% were diagnosed with endocarditis. Overall, 35.9% of the patients were assessed with echocardiography (Table 2). Among those with no ascertained focus of infection, 29.8% were examined with echocardiography. The mortality varied by focus of infection. The highest 30-day mortality risk was seen among those with a respiratory focus (42.4%, 95% CI 26.0-58.5%) and unknown focus of infection (38.7%, 95% CI 27.5-48.2%) (Table 4). The highest 90-day mortality risk was seen for the same foci (Additional file 1: Table S2). The lowest 30-day risk was seen for urinary tract focus (11.2%, 95% CI 3.4-24.0%), abscess (15.6%, 96% CI 4.3-35.4%), and osteomyelitis/septic arthritis (13.8%, 95% CI 4.9-26.1%) (Table 4). For these foci there was also a low risk at 90 days (Additional file 1: Table S2). The mortality risk for patients with endocarditis was low at 30 days (13.4%, 95% CI 2.8-37.4%) (Table 4), but the risk increased nearly three-fold to 39.3% at 90 days (95% CI 17.0-63.7%) (Additional file 1: Table S2).

Overall, 24.7% of the patients were treated in the intensive care unit or a medical high dependency unit. Among those with septic shock 79.2% were admitted to the ICU or medical high dependency unit, whereas 30.6% of those with severe sepsis received care at the ICU or medical high dependency unit. The risk of acquiring severe sepsis/septic shock increased with age from 30.7% (95% CI 21.7-41.6%) for those aged < 60 to 49.0% (95%CI 40.3-57.7%) for those aged ≥80. Among individual comorbidities the risk of severe sepsis or septic shock was highest for those with heart failure (67.4%, 95% CI 51.2-80.6%), renal failure (60.5%, 95% CI 45.3-73.9) and chronic pulmonary disease (55.6%, 95% CI 43.0-67.6%). The infectious foci associated with the highest risk of developing severe disease included endocarditis (86.0%, 95% CI 64.4-95.8%), respiratory focus (52.4%, 95% CI 36.2-68.1%) and unknown infectious foci (51.0%, 95% CI 40.6-61.3%) (Additional file 1: Table S3).

The most common empirical antibiotic regimens administered were penicillin combined with an aminoglycoside (17.3%), cefuroxime-containing regimens (16.9%) and dicloxacillin-containing regimens (16.6%). 2.9% received no initial treatment. 15.3% received initial treatment that was not effective against the microbe in question, and most of these were treated with penicillin in monotherapy. As definitive treatment, 51% of the patients were treated with dicloxacillin-containing regimens. 2.7% received no definitive treatment and 2.9% received inefficient definitive treatment (Table 2). Among those with community-acquired infection, 31 patients (28.7%) received inefficient or no treatment initially. The same was true for 16.7% of those with healthcare-acquired infection and 10.1% of those with hospital-acquired infection. 119 patients, (31.9%) received surgical treatment including drainage of abscesses and pleural fluid, wound revisions, amputations, arthrocentesis, removal of orthopedic hardware, stenting of the renal pelvis or the common bile duct and cardiac valve surgery. The median duration of intravenous antibiotic therapy was 10 days and the meadian total duration including oral antibiotic treatment was 14 days (Table 2). When examining the first (1996–2003) and second (2004–2011) halves of the study separately, 30.6% of the patients in the first period and 32.8% of the patients in the second period received surgical treatment. 32.6% of the patients in the first period were examined with echocardiography versus 38.0% in the second period of the study.

This study was carried out over a period of 16 years. The age, sex and comorbidity-adjusted 30-day mortality risk did not differ between the first and second halves of the study period with an adjusted mortality risk of 27.3% (95% CI 18.1-33.1%) for 1996–2003 versus 27.4% (95% CI 19.4-31.4%) for 2004–2011 (Table 4). The same pattern was seen for 90-day adjusted mortality risk (Additional file 1: Table S2).

## Discussion

This study highlights several important aspects regarding bloodstream infection and sepsis with *Staphylococcus aureus*. Only 6 patients out of 379 with positive blood cultures did not meet the criteria for sepsis, emphasizing the clinical relevance of a positive blood culture for *Staphylococcus aureus*. Our findings also confirm that the proportion of *Staphylococcus aureus* bloodstream infections caused by MRSA in Norway is low. There was a strong influence of age, comorbid burden, disease severity and focus of infection on the outcome, and there was a stable high case fatality rate of 27.3% during the study period. Our 30 day all-cause mortality rate is higher than in other Scandinavian studies [7,26], but a clinical study of *Staphylococcus aureus* bacteremia in inpatients in Oxfordshire and one German study found similar mortality rates [27,28]. Some of these differences could be due to differences in study design and patient characteristics. One factor contributing to the high mortality rate in our population could be the old age of our patients. We also found a clear association between comorbid diseases and mortality, and a high proportion of our patients suffered from comorbid disease. The association between an increased CCI and mortality in patients with *Staphylococcus aureus* bacteremia has been shown earlier by Lesens et al. [29].

A high proportion of our patients suffered severe sepsis or septic shock during their episode, with a striking accompanying increase in mortality even when adjusting for age, sex and comorbid burden. This strong association has been observed in several other studies [13]. The same pattern was seen for severity classified by the Pitt bacteremia score, with increased mortality for patients with a score of 2 or more. Pitt bacteremia score has been shown to be significantly correlated with mortality both in prospective clinical and ICU based trials [30,31]. In our cohort, nearly 70% of those with severe sepsis and 20% of those with septic shock were treated on a general hospital ward. Although care at a regular bed unit can be of very high quality and there can be various good reasons for not increasing the level of care for example a palliative setting, this still highlights the importance of well-established routines to identify and follow up these patients and ensure the best standard of care.

Assigning focus of infection of *Staphylococcus aureus* is challenging because of the organism’s affinity to foreign objects and its propensity to generate metastatic foci [3]. We found a high mortality among patients with an unknown focus of infection. This is in line with results from previous studies [8,32]. The importance of ascertaining a focus of infection and removing it if possible has been well established [19]. We found lower mortality among those with bone and joint infection, abscess and primary urinary focus. The two latter can possibly be explained by the possibility to promptly remove the focus of infection in most cases. Bacteriuria accompanying *Staphylococcus aureus* bloodstream infection has been seen as a sign of hematogenous spread of infection with higher mortality, but ascending infection is also possible and has been linked to a more favorable outcome [6,24,33,34]. Surprisingly, we also found a low 30-day mortality of endocarditis of 13.4% in our patient cohort, but this was increased by a three-fold by day 90 to 39.3%. We do not have a definite explanation for this difference in short and longer term mortality, but more intensive follow-up and management in the initial phases of the disease as well as ongoing damage to valves and endocardial structures may be contributing factors. Overall, we had a low proportion of endocarditis of 4.8% in our cohort. This is lower than the 8.3% found by Kaasch et al. in a recent pooled analysis of 5 prospective observational studies [8] and much lower than a recent multicenter study in Denmark where 22% of the patients screened with echocardiography were diagnosed with endocarditis [35]. One possible explanation of the low prevalence in our cohort is underdiagnosis due to the low rate of examination with echocardiography. Rasmussen et al. identified an unknown focus of infection as one of several independent risk factors for infective endocarditis in patients with *Staphylococcus aureus* bacteremia, and only 29.8% of our patients with unknown focus were examined with echocardiography. It is possible that some of the patients without ascertained focus of infection had endocarditis. Underdiagnosis of endocarditis may lead to inadequate management including antibiotic treatment, and may be one factor explaining the rather high case-fatality rate in this study. The higher case-fatality rate among those with an unknown focus of infection may be due to a reluctance to carry out investigations on patients where the overall prognosis is unfavorable, such as end-of life situations. On the other hand the low performance of echocardiography and the increased case-fatality rate may also indicate that fairly simple improvements in clinical standards of assessment may help strengthen the diagnostic accuracy, treatment and hopefully outcome for this patient group.

The prevalence of MRSA in this cohort was low, and most patients received adequate antibiotic management, especially after the microbe was identified. Interestingly, nearly a third of those with community-acquired infection received inadequate therapy, often penicillin in monotherapy. This is probably a sign that the level of suspicion for this pathogen is lower than for patients that contract this infection in a healthcare-associated setting. Delay of adequate treatment is of clinical importance because of the associated increased risk of infection-related mortality [36]. There were standardized local procedures available for treating sepsis with an unknown microbe and focus, primarily recommending the use penicillin and an aminoglycoside such as gentamicin, tobramicin or netilmicin. There was no standardized guideline available for the management of *Staphylococcus aureus* bloodstream infection specifically, but national guidelines recommended dicloxacillin as the preferred antibiotic to treat *Staphylococcus aureus* infections [37]. The management of the patients and choice of treatment was at the discretion of the managing physician, as was the decision of referral to an infectious disease specialist. There was no specific local guideline recommending echocardiography for patients with *Staphylococcus aureus* bloodstream infections, but the need for echocardiography in these patients was emphasized during regular internal teaching sessions when bloodstream infection or endocarditis was being reviewed.

Our study has several limitations. It is carried out in a single Norwegian region and the results are not necessarily representative for other geographic areas. We do however believe that it could be representative of the situation in many other local hospitals of similar size. The positive blood cultures were prospectively registered by our microbiology laboratory, but the clinical information was retrieved from the hospital records at a later date, which is inferior to standardized prospective clinical registration. The assignment of focus of infection was largely based on clinical assessments and investigations carried out at the time of patient admission. The investigations were carried out at the discretion of the treating clinician, and as such the diagnostic accuracy may have varied between patients. However, this data reflects the clinical everyday management and decision basis that were available for these patients. All the data were not available for all the patients, especially during the earlier part of the study and regarding antibiotic management. However, this design confers a greater level of detail and accuracy than observational studies based on discharge data.

## Conclusions

Despite great efforts to improve survival of sepsis the last decade, there was no reduction in case fatality rate during the study period. There have been some promising results both of infectious disease consultation of all patients with *Staphylococcus aureus* bacteremia [38-40] and implementation of Quality of Care bundles for the management of these patients [41,42]. These types of initiatives along with improved measures of prevention, diagnosis and management will hopefully help improve the outcome of *Staphylococcus aureus* bloodstream infection in the future.

## Additional file

Additional file 1: Table S1. 90-day mortality in relation to patient characteristics prior to infection. **Table S2.** 90-day mortality in relation to disease acquisition, severity, focus and time period. **Table S3.** Risk of severe sepsis/septic shock according to prior patient characteristics and infection-related characteristics.
