# Supplementary material for: Epidemiology and outcome of Staphylococcus aureus bloodstream infection and sepsis in a Norwegian county 1996–2011: an observational study
Source: BMC Infect Dis. 2015 Mar 4;15:116. doi: 10.1186/s12879-015-0849-4 (PMC4351681; doi:10.1186/s12879-015-0849-4)
Supplement: Additional file 1: Table S1. — 90-day mortality in relation to patient characteristics prior to infection. Table S2. 90-day mortality in relation to disease acquisition, severity, focus and time period. Table S3. Risk of severe sepsis/septic shock according to prior patient characteristics and infection-related characteristics. [file 12879_2015_849_MOESM1_ESM.docx]

Supplementary tables

**Table S1: 90-day mortality in relation to patient characteristics prior to infection**

|  |  | Age- and sex-adjusted | | | | | |
| --- | --- | --- | --- | --- | --- | --- | --- |
| Characteristics | No. of deaths within 90 days | 90-day mortality within category  (%) | Odds ratio | 95% CI | p | Mortality risk (%) | 95% CI |
| **Age (years)** |  |  |  |  |  |  |  |
| <60 | 15 | 18.5 | 1 | Reference |  | 18.6 | 11.5-28.6 |
| 60-69 | 19 | 32.8 | 2.14 | 0.97-4.69 | 0.06 | 32.8 | 22.0-45.8 |
| 70-79 | 41 | 37.6 | 2.65 | 1.34-5.24 | 0.005 | 37.7 | 29.1-47.1 |
| ≥80 | 60 | 48.0 | 4.00 | 2.06-7.75 | <0.001 | 47.7 | 39.1-56.5 |
| p for trend |  |  |  |  | <0.001 |  |  |
| **Sex** |  |  |  |  |  |  |  |
| Male | 76 | 33.8 | 1 | Reference |  | 34.3 | 27.3-40.0 |
| Female | 59 | 39.9 | 1.23 | 0.79-1.92 | 0.35 | 39.0 | 30.5-46.5 |
| **Charlson Comorbidity Index (CCI)** |  |  |  |  |  |  |  |
| 0 | 15 | 17.6 | 1 | Reference |  | 18.6 | 10.6-27.3 |
| 1-2 | 50 | 31.3 | 1.96 | 1.01-3.83 | 0.05 | 30.4 | 22.6-37.0 |
| ≥3 | 70 | 54.7 | 5.72 | 2.87-11.4 | <0.001 | 54.5 | 45.5-63.5 |
| p for trend |  |  |  |  | <0.001 |  |  |
| **Comorbidities $** |  |  |  |  |  |  |  |
| Malignant disease | 44 | 45.8 | 1.77 | 1.08-2.89 | 0.02 | 45.6 | 35.2-55.6 |
| Renal failure | 22 | 48.9 | 1.75 | 0.91-3.36 | 0.09 | 47.3 | 32.5-62.0 |
| Diabetes mellitus | 30 | 41.7 | 1.21 | 0.70-2.08 | 0.49 | 39.6 | 28.0-50.7 |
| Hypertension | 45 | 39.5 | 1.00 | 0.62-1.61 | 0.99 | 36.1 | 26.6-44.7 |
| Cardiovascular disease | 64 | 41.6 | 1.11 | 0.70-1.77 | 0.65 | 37.5 | 29.0-45.1 |
| Heart failure | 23 | 57.5 | 2.03 | 1.03-4.02 | 0.04 | 50.6 | 34.8-66.1 |
| Chronic pulmonary disease | 30 | 48.4 | 1.73 | 0.98-3.05 | 0.06 | 46.5 | 33.8-58.9 |
| Rheumatic disease | 17 | 43.6 | 1.44 | 0.72-2.90 | 0.31 | 43.5 | 28.0-59.3 |

$ Those not having the condition in question were used as reference category for each individual comorbidity studied in this analysis.

**Table S2: 90-day mortality in relation to disease acquisition, severity, focus and time period**

|  |  |  | Age-, sex- and comorbidity-adjusted | | | | |
| --- | --- | --- | --- | --- | --- | --- | --- |
| Characteristic | No. of deaths within 90 days | 90-day mortality within category (%) | Odds ratio | 95% CI | p | Mortality risk (%) | 95% CI |
| **Place of acquisition** |  |  |  |  |  |  |  |
| Community acquired | 31 | 28.7 | 1 | Reference |  | 35.5 | 23.7-43.9 |
| Health-care associated | 61 | 39.1 | 0.92 | 0.51-1.68 | 0.79 | 33.9 | 23.9-39.6 |
| Hospital acquired | 43 | 39.4 | 1.27 | 0.68-2.39 | 0.45 | 40.4 | 29.2-48.8 |
| **Severity** |  |  |  |  |  |  |  |
| Sepsis without organ failure | 47 | 22.0 | 1 | Reference |  | 23.2 | 15.0-26.4 |
| Severe sepsis | 55 | 49.5 | 3.72 | 2.18-6.37 | <0.001 | 48.7 | 38.5-58.4 |
| Septic shock | 33 | 68.8 | 7.45 | 3.52-15.75 | <0.001 | 63.2 | 49.2-78.4 |
| p for trend |  |  |  |  | <0.001 |  |  |
| **Pitt bacteremia score** |  |  |  |  |  |  |  |
| 0 | 35 | 27.6 | 1 | Reference |  | 27.5 | 17.4-33.2 |
| 1 | 34 | 29.6 | 1.18 | 0.64-2.17 | 0.59 | 30.5 | 19.7-37.2 |
| 2 | 28 | 43.1 | 2.54 | 1.28-5.05 | 0.008 | 45.9 | 32.5-58.3 |
| ≥3 | 38 | 57.6 | 3.48 | 1.77-6.82 | <0.001 | 52.6 | 39.6-65.8 |
| p for trend |  |  |  |  | <0.001 |  |  |
| **Focus of infection** |  |  |  |  |  |  |  |
| Unknown | 48 | 51.1 | 1 | Reference |  | 46.3 | 34.8-56.6 |
| Respiratory focus | 19 | 51.4 | 1.31 | 0.57-3.01 | 0.52 | 52.1 | 35.3-68.7 |
| Urinary tract | 7 | 21.2 | 0.22 | 0.08-0.60 | 0.003 | 18.7 | 6.9-31.6 |
| Skin/soft tissue | 30 | 41.1 | 0.74 | 0.38-1.45 | 0.38 | 39.9 | 27.0-50.8 |
| Abscess | 4 | 14.1 | 0.24 | 0.07-0.83 | 0.025 | 19.9 | 6.0-39.1 |
| Intravenous catheter | 8 | 28.6 | 0.34 | 0.13-0.92 | 0.03 | 25.3 | 10.5-41.0 |
| Endocarditis | 6 | 33.3 | 0.72 | 0.22-2.3 | 0.58 | 39.3 | 17.0-63.7 |
| Osteomyelitis/Septic arthritis | 6 | 13.6 | 0.21 | 0.08-0.58 | 0.03 | 18.0 | 6.7-30.2 |
| Other focus | 7 | 36.8 | 0.79 | 0.26-2.36 | 0.67 | 41.2 | 19.4-64.2 |
| **Time period** |  |  |  |  |  |  |  |
| 1996-2003 | 46 | 31.9 | 1 | Reference |  | 35.9 | 25.7-42.4 |
| 2004-2011 | 89 | 38.9 | 1.02 | 0.63-1.65 | 0.92 | 36.4 | 27.7-41.1 |

**Table S3: Risk of severe sepsis/septic shock according to prior patient characteristics and infection-related characteristics**

|  |  |  | Age-, sex- and comorbidity*-adjusted | | | | |
| --- | --- | --- | --- | --- | --- | --- | --- |
| Characteristic | No with severe sepsis or septic shock | Severe sepsis or septic shock in category (%) | Odds ratio | 95% CI | p | Risk of severe sepsis or septic shock (%) | 95% CI |
| **Age (years)** |  |  |  |  |  |  |  |
| <60 | 25 | 30.9 | 1 | Reference |  | 30.7 | 21.7-41.6 |
| 60-69 | 24 | 41.4 | 1.59 | 0.78-3.21 | 0.20 | 41.3 | 29.4-54.3 |
| 70-79 | 49 | 45.0 | 1.83 | 1.00-3.36 | 0.05 | 44.9 | 35.8-54.3 |
| ≥80 | 61 | 48.8 | 2.16 | 1.20-3.90 | 0.01 | 49.0 | 40.3-57.7 |
| p for trend |  |  |  |  | 0.01 |  |  |
| **Sex** |  |  |  |  |  |  |  |
| Male | 98 | 43.6 | 1 | Reference |  | 43.9 | 37.3-50.4 |
| Female | 61 | 41.2 | 0.88 | 0.57-1.34 | 0.54 | 40.7 | 32.8-48.7 |
| **Charlson Comorbidity Index** |  |  |  |  |  |  |  |
| 0 | 32 | 37.6 | 1 | Reference |  | 39.5 | 29.3-50.3 |
| 1-2 | 67 | 41.9 | 1.1 | 0.64-1.91 | 0.73 | 41.9 | 34.2-49.6 |
| ≥3 | 60 | 46.9 | 1.29 | 0.72-2.29 | 0.39 | 45.6 | 36.8-54.4 |
| p for trend |  |  |  |  | 0.38 |  |  |
| **Comorbidities $** |  |  |  |  |  |  |  |
| No underlying illness | 5 | 25.0 | 0.52 | 0.18-1.48 | 0.22 | 28.5 | 12.3-52.4 |
| Malignant disease | 33 | 34.4 | 0.60 | 0.37-0.97 | 0.04 | 33.7 | 24.6-43.4 |
| Renal failure | 28 | 62.2 | 2.30 | 1.19-4.43 | 0.01 | 60.4 | 45.3-73.9 |
| Diabetes mellitus | 32 | 44.4 | 1.04 | 0.62-1.76 | 0.87 | 43.5 | 32.3-55.0 |
| Hypertension | 50 | 43.9 | 0.99 | 0.62-1.57 | 0.97 | 42.5 | 33.3-51.8 |
| Cardiovascular | 67 | 43.5 | 0.84 | 0.54-1.32 | 0.46 | 40.3 | 32.2-48.3 |
| Heart failure | 28 | 70.0 | 3.19 | 1.55-6.60 | 0.002 | 67.4 | 51.2-80.6 |
| Chronic pulmonary disease | 35 | 56.5 | 1.90 | 1.08-3.32 | 0.03 | 55.6 | 43.0-67.6 |
| Rheumatic disease | 15 | 38.5 | 0.85 | 0.43-1.71 | 0.66 | 39.3 | 24.9-55.3 |
| **Place of acquisition** |  |  |  |  |  |  |  |
| Community acquired | 46 | 42.6 | 1 | Reference |  | 45.7 | 35.9-55.5 |
| Health-care associated | 72 | 46.2 | 0.92 | 0.54-1.56 | 0.75 | 43.6 | 35.5-51.6 |
| Hospital acquired | 41 | 37.6 | 0.73 | 0.41-1.29 | 0.28 | 38.2 | 29.2-47.5 |
| **Focus of infection** |  |  |  |  |  |  |  |
| Unknown | 50 | 53.2 | 1 | Reference |  | 51.0 | 40.6-61.3 |
| Respiratory focus | 19 | 51.4 | 1.06 | 0.48-2.33 | 0.89 | 52.4 | 36.2-68.1 |
| Urinary tract | 13 | 39.4 | 0.54 | 0.24-1.25 | 0.15 | 36.6 | 21.5-54.0 |
| Skin/soft tissue | 24 | 32.9 | 0.46 | 0.24-0.87 | 0.02 | 32.6 | 22.4-43.9 |
| Abscess | 6 | 22.2 | 0.33 | 0.12-0.92 | 0.04 | 26.1 | 11.9-46.6 |
| Intravenous catheter | 9 | 32.1 | 0.47 | 0.19-1.20 | 0.12 | 33.5 | 17.8-53.0 |
| Endocarditis | 15 | 83.3 | 6.18 | 1.61-23.73 | 0.008 | 86.0 | 64.4-95.8 |
| Bone/joint | 12 | 27.3 | 0.35 | 0.15-0.79 | 0.012 | 27.0 | 15.3-42.0 |
| Other focus | 11 | 57.9 | 1.38 | 0.50-3.85 | 0.54 | 58.8 | 36.1-78.5 |
| **Time period** |  |  |  |  |  |  |  |
| 1996-2003 | 62 | 43.1 | 1 | Reference |  | 44.3 | 36.1-52.5 |
| 2004-2011 | 97 | 42.4 | 0.89 | 0.58-1.38 | 0.61 | 41.6 | 35.0-48.0 |

*The association between underlying comorbidities and the risk of severe sepsis or septic shock are only adjusted for age and sex.

$ Those not having the condition in question were used as reference category for each individual comorbidity studied in this analysis.
